# Thigh-worn accelerometry: a comparative study of two no-code classification methods for identifying physical activity types

**DOI:** 10.1186/s12966-024-01627-1

**Published:** 2024-07-17

**Authors:** Claas Lendt, Theresa Braun, Bianca Biallas, Ingo Froböse, Peter J. Johansson

**Affiliations:** 1 Institute for Movement Therapy and Movement-Oriented Prevention and Rehabilitation, German Sport University Cologne, Cologne, Germany; 2 Occupational and Environmental Medicine, Uppsala University Hospital, Uppsala, Sweden; 3 Department of Medical Sciences, Occupational and Environmental Medicine, Uppsala University, Uppsala, Sweden

**Keywords:** Accelerometer, Physical behaviour, Sedentary behaviour, Activity classification, Human activity recognition, Validation

## Abstract

**Background:**

The more accurate we can assess human physical behaviour in free-living conditions the better we can understand its relationship with health and wellbeing. Thigh-worn accelerometry can be used to identify basic activity types as well as different postures with high accuracy. User-friendly software without the need for specialized programming may support the adoption of this method. This study aims to evaluate the classification accuracy of two novel no-code classification methods, namely SENS motion and ActiPASS.

**Methods:**

A sample of 38 healthy adults (30.8 ± 9.6 years; 53% female) wore the SENS motion accelerometer (12.5 Hz; ±4 g) on their thigh during various physical activities. Participants completed standardized activities with varying intensities in the laboratory. Activities included walking, running, cycling, sitting, standing, and lying down. Subsequently, participants performed unrestricted free-living activities outside of the laboratory while being video-recorded with a chest-mounted camera. Videos were annotated using a predefined labelling scheme and annotations served as a reference for the free-living condition. Classification output from the SENS motion software and ActiPASS software was compared to reference labels.

**Results:**

A total of 63.6 h of activity data were analysed. We observed a high level of agreement between the two classification algorithms and their respective references in both conditions. In the free-living condition, Cohen’s kappa coefficients were 0.86 for SENS and 0.92 for ActiPASS. The mean balanced accuracy ranged from 0.81 (cycling) to 0.99 (running) for SENS and from 0.92 (walking) to 0.99 (sedentary) for ActiPASS across all activity types.

**Conclusions:**

The study shows that two available no-code classification methods can be used to accurately identify basic physical activity types and postures. Our results highlight the accuracy of both methods based on relatively low sampling frequency data. The classification methods showed differences in performance, with lower sensitivity observed in free-living cycling (SENS) and slow treadmill walking (ActiPASS). Both methods use different sets of activity classes with varying definitions, which may explain the observed differences. Our results support the use of the SENS motion system and both no-code classification methods.

**Supplementary Information:**

The online version contains supplementary material available at 10.1186/s12966-024-01627-1.

## Background

Physical behaviour, encompassing both physical activity and sedentary behaviour, is recognized as a significant modifiable determinant of health. These behaviours are characterized by variables such as intensity, duration, and type, and are known to influence various health outcomes [1–3]. Consequently, the development of accurate and user-friendly methods for the assessment of physical behaviour in health-related research and practice is pivotal. Such methods are instrumental for establishing further associations with health outcomes, evaluating intervention efficacy, and implementing large-scale health monitoring [4, 5].

Recent advancements in wearable accelerometer technology have facilitated the objective assessment of physical behaviour in free-living conditions [6]. The placement of these sensors on the body significantly impacts the information embedded in the acceleration signal, thereby affecting the subsequent data processing and analysis capabilities. Thigh-worn accelerometers are increasingly being used in large cohort studies to study 24-hour physical behaviour patterns [4, 7]. The 3D acceleration signal can be used to calculate the inclination and acceleration of the thigh over a given time interval. Using validated activity classification algorithms, this information can be used to differentiate selected physical activity types and postures, such as sitting, standing, walking or cycling, with very high accuracy [5, 8–11].

However, the implementation of thigh-worn accelerometers in research settings often presents challenges, particularly in terms of sensor attachment and data processing, which typically require specialized knowledge. Recent research efforts offer some improvements regarding standardisation [5, 12, 13]. A recent study indicated that health-related researchers using accelerometers often find code-based analysis to be too complicated to use [14]. Thus, no-code or low-code classification methods can support the use of objective measurement of physical activity types. Consideration of key aspects such as ease of use, high standardisation and simple processing of sensor data can be considered crucial for the broader adoption of thigh-worn assessment methods in future studies [13].

The ActiPASS software was developed using the validated Acti4 algorithm [5, 8, 10] and integrates various methods for data processing, such as automatic calibration and reference identification [15]. ActiPASS allows the import and processing of different data formats used by various common accelerometer brands (e.g. Axivity, Actigraph, activPAL). The software encompasses a graphical user interface to allow a user-friendly interaction. Several outputs can be used for further analysis, such as the export of 1-second activity classification data or summary tables.

The SENS motion accelerometer system (SENS Innovation ApS, Copenhagen, Denmark), comprising a thigh-worn accelerometer, adhesive patch and accompanying software is another notable development in the field of thigh-worn accelerometry. While being relatively new, the accelerometer system is already used as part of a national surveillance study on 24-hour physical behaviour [16]. The hardware of the accelerometer system represents an improvement over most commonly used accelerometer types, as it allows for wireless communication with a smartphone app and thus can enable fully remote data collection. In a research context, this can potentially help to reduce the burden for researchers and participants, decrease the time needed for data collection and increase adherence rates for large-scale studies [13].

The system additionally includes a web application which allows users to automatically perform activity classification and data export. Previous studies have investigated the validity of the system in clinical populations and under standardised laboratory conditions [17–19]. However, the systems classification accuracy in healthy adult populations and free-living conditions remains unknown. This study aims to address this gap by assessing the accuracy of the SENS motion system in classifying basic physical activity types and sedentary behaviour in healthy adults. Additionally, this research seeks to compare the performance of the SENS motion system’s classification algorithm with that of the validated algorithms used in the ActiPASS software.

## Methods

### Participants

Thirty-eight healthy adults (53% female, age 30.8 ± 9.6 years; body mass index 23.7 ± 3.1 kg/m²) were recruited via newsletters and posters at the university campus. Participants were eligible if they were between 18 and 59 years, free from any known acute or chronic orthopaedic, neurological, or cardiovascular illness and otherwise capable of performing unrestricted physical activity.

We performed a simulation based on previous research [9] to determine the necessary sample size. Cohen’s Kappa values for the sample population were drawn from a continuous uniform distribution with a range of 0.60 to 1. In total, *n* = 1.000 simulations were performed for each respective sample size. Based on the results from the simulations the necessary sample size was found to be approximately 34 subjects to achieve a mean standard error smaller than 0.02.

### Instrumentation

**Fig. 1:**
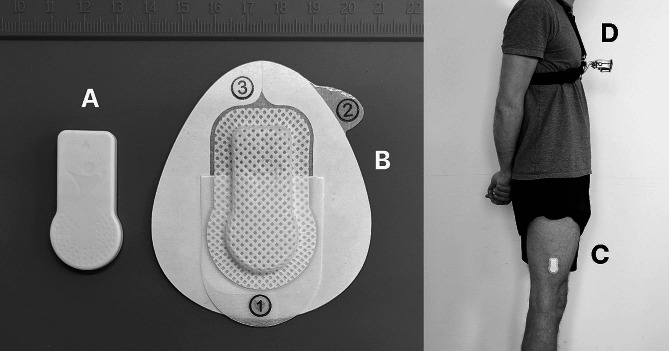
The SENS motion sensor (**A**) and adhesive patch (**B**). The SENS motion sensor is attached to the right lateral thigh (**C**). During free-living condition, the camera is mounted to the chest of the participant (**D**)

The SENS motion system comprises a small-scale and lightweight triaxial accelerometer (47 × 22 × 4.5 mm, 7 g), a smartphone application for wireless data transfer and a browser-based web application for sensor management, data analysis and data export. The accelerometer has a fixed sample rate of 12.5 Hz with a measurement range of ± 4 g and can be attached to the skin using an adhesive patch (Fig. 1). The waterproof sensor is to be placed on the lateral side of the thigh above the knee.

### Laboratory protocol

Data collection encompassed a single visit to the laboratory at the university campus for approximately 2.5 h. Upon arrival, each participant signed the informed consent form including data privacy statement and answered the Physical Activity Readiness Questionnaire [20] to ensure inclusion criteria were met. Subsequently, the sensor was placed 10 cm above the lateral epicondyle of the femur of the right leg using the manufacturer’s adhesive patch (Fig. 1). A total of five individual sensors were used in the study and one sensor was randomly selected for each participant from this pool of sensors.

A semi-standardised activity protocol with six activities and 14 conditions was performed by each participant (Table 1). The protocol was designed to simulate everyday activities, different intensities, and body positions during a typical day. Each participant was instructed to mimic free-living behaviour as much as possible. During standing and sitting conditions, participants were placed at a height adjustable desk and had to solve a series of Sudoku puzzles. Walking and running were performed on an instrumented treadmill (quasar med, h/p/cosmos, Nussdorf-Traunstein, Germany) and cycling was performed on a stationary cycling ergometer (ergoselect 100, Ergoline, Bitz, Germany). For the slow running condition, participants were specifically instructed to perform running motions instead of fast walking.

**Table 1:** Standardized activity protocol used for the laboratory condition

Activity type	Description	Duration (minutes)
Standing	Upright standing at a height-adjusted desk.	5
Sitting	Sitting on a chair at a height-adjusted desk.	
Lying (supine)	Lying down in supine position with or without head on a foam pillow	3
Lying (side)	Lying down on the left side with or without head on a foam pillow	
Lying (prone)	Lying down in prone position with or without head on a foam pillow	
Walking (slow)	Walking on treadmill with 0.5 m/s	
Walking (moderate)	Walking on treadmill with 0.8 m/s	
Walking (fast)	Walking on treadmill with 1.2 m/s	
Running (slow)	Running on treadmill with 1.8 m/s	
Running (moderate)	Running on treadmill with 2.3 m/s	
Running (fast)	Running on treadmill with 2.8 m/s	
Cycling (slow)	Cycling with 50 Watts and 40 rpm	
Cycling (moderate)	Cycling with 75 Watts and 60 rpm	
Cycling (fast)	Cycling with 100 Watts and 80 rpm	

m/s = meters per second; rpm = revolutions per minute

### Free-living protocol

Participants were equipped with a video camera (Hero 4 Black Edition, GoPro, San Mateo, USA) to record their activities during the free-living protocol. The camera was placed on the participants chest using a chest harness. The camera was positioned to point down at the feet to allow recording of the lower extremities (Fig. 1). The accelerometer data and video data were time-synchronised using a motion-based marker. After a 15-second period of rest, each participant dropped onto their heels three times from a toe stand, followed by another 15-second period of rest. Following the synchronisation procedure, participants left the laboratory and were free to engage in any activity on and off the university campus for approximately 60 min. Participants were encouraged to engage in a variety of physical activities during the free-living condition. Where possible, participants were specifically encouraged to include cycling on their own bicycle in their activities. Participants then returned to the laboratory where the sensor and camera were collected.

### Data processing

Processing of all tabular data was carried out using R Statistical Software (v4.3.2) [21]. A Shiny application [22] was developed in R to manually log the onset of each activity during the laboratory condition. Labelled timestamps from the Shiny app were used to create reference data for the laboratory condition. The accelerometer data were time synchronized with the Shiny app by quickly flipping over the accelerometer on a level surface and manually identifying the change in acceleration and the respective timestamp within the acceleration signal. We manually identified the heel drops and their respective timestamps in the video and raw acceleration data for time synchronization of the free-living data.

**Fig. 2:**
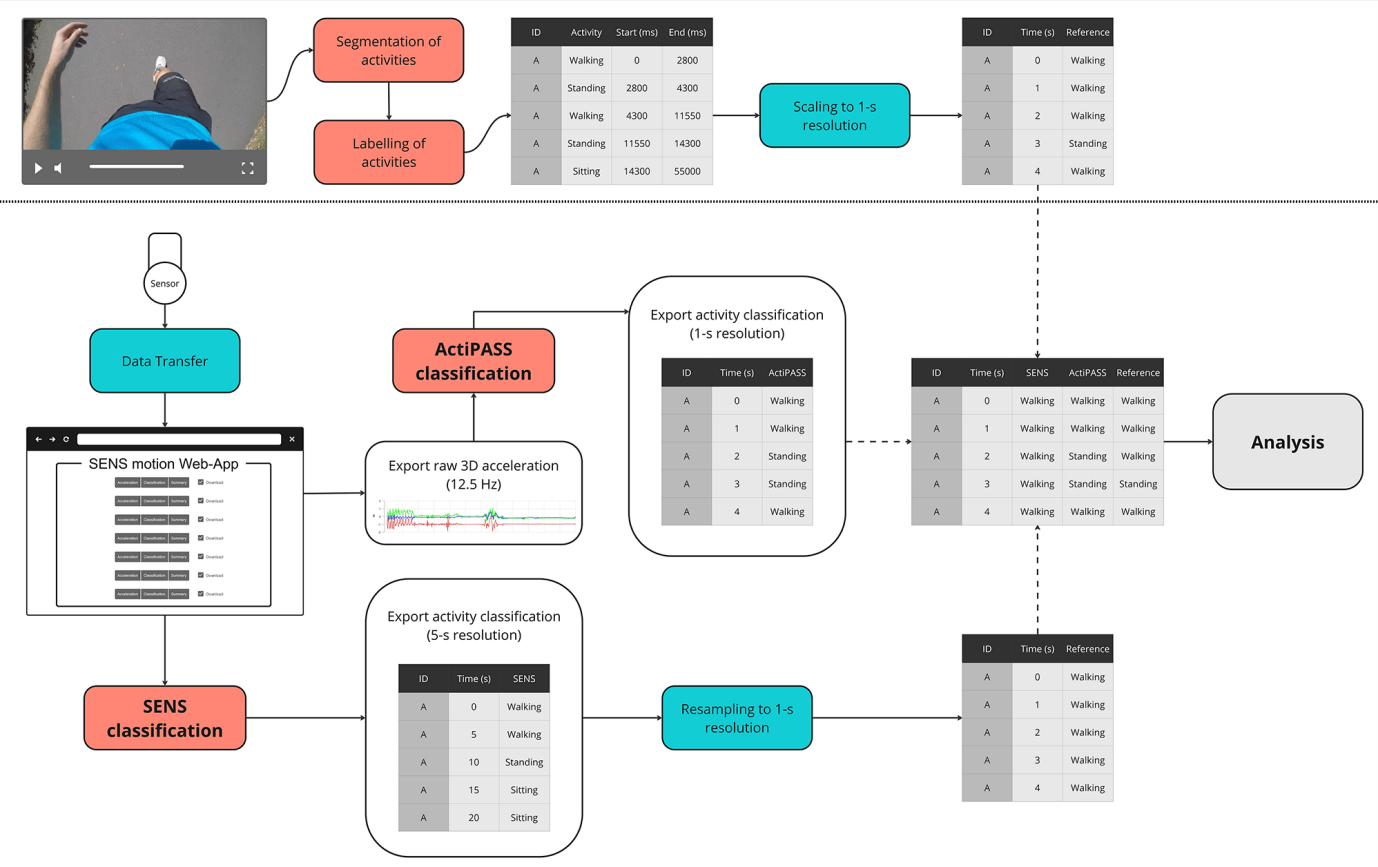
Processing pipeline for the video annotation and activity classification using the SENS web-application and ActiPASS software

After data transfer, SENS activity classification is performed automatically on the raw acceleration data without the need for any user interaction. The acceleration data is classified in non-overlapping 5-second intervals. The resulting classification data were exported and downloaded in CSV format using the web application. The data were subsequently resampled by segmenting each 5-second interval into five individual 1-second intervals to allow a direct comparison with the reference and ActiPASS classifications. Additionally, raw triaxial accelerometer time-series data (12.5 Hz) was exported in binary file format (Fig. 2). The binary files were then imported into ActiPASS (Version 1.58 [15]) for activity classification with default ProPASS mode settings. These include auto-calibration and auto-identification of the individual reference position [15, 23]. The ActiPASS classification is based on the validated and open-source Acti4 algorithm and uses 2-second intervals with a 50% overlap as well as a mode-based filtering approach. The detailed design of the algorithm has been previously published [8, 24]. ActiPASS classification results were exported in CSV format in 1-second intervals. SENS and ActiPASS activity labels were further grouped into five primary physical activity types encompassing sedentary, standing, walking, running, and cycling (Additional file 1, Table S1). The grouping was performed to allow direct comparison between the algorithms output.

Videos were recorded at 30 frames per second and 1920 × 1080 pixel resolution. Videos were automatically saved as consecutive 20-minute segments in an MP4 format on a 64 GB microSD memory card. Video data were downloaded from the wearable camera using associated software (GoPro Quik, Version 2.7.0.945). Video segments for each participant were subsequently merged using open-source video editing software (Shotcut, Version 22.12.21). The videos were annotated following a coding scheme, which was adapted from a previous study [9] (Additional file 1, Table S2). Activities were labelled as undefined if they did not fit any of the activity labels.

Video data were annotated by a single rater using ELAN (Version 6.4) open-source video annotation software [25]. First, videos were segmented on a frame-by-frame resolution into individual activities using the software’s segmentation mode to identify the exact start and end of each activity. Each activity was then reviewed and labelled according to the coding scheme using the annotation mode. Subsequently, start and end times of each activity were rounded to the nearest full second to match the 1-second resolution of the activity classifications. The annotated video data were used as reference for the free-living condition. Five videos were randomly selected and subsequently annotated by a second independent rater to quantify the interrater agreement.

### Statistical analysis

The classification performance metrics for SENS motion and ActiPASS included precision, sensitivity, specificity, and balanced accuracy:

- Precision was calculated as the sum of true positives divided by the sum of true and false positives.
- Sensitivity was calculated by dividing the sum of true positives by the sum of true positives and false negatives.
- Specificity was calculated as the sum of true negatives divided by the sum of true negatives and false positives.
- Balanced accuracy was calculated as the mean of sensitivity and specificity.

Cohen’s Kappa [26] was calculated to quantify overall agreement between SENS motion and ActiPASS algorithms with the respective reference using the irr-package [27]. Cohen’s Kappa was further calculated to quantify the level of agreement between the two raters for the video annotation.

## Results

All (*n* = 38) participants performed each activity in the laboratory protocol except one who did not complete the last running condition. Cycling data for one participant was excluded due to sensor detachment. We further excluded free-living data due to sensor detachment (*n* = 2) and insufficient lightning conditions (*n* = 1). For the free-living condition, data from *n* = 35 participants were used for analysis.

**Fig. 3:**
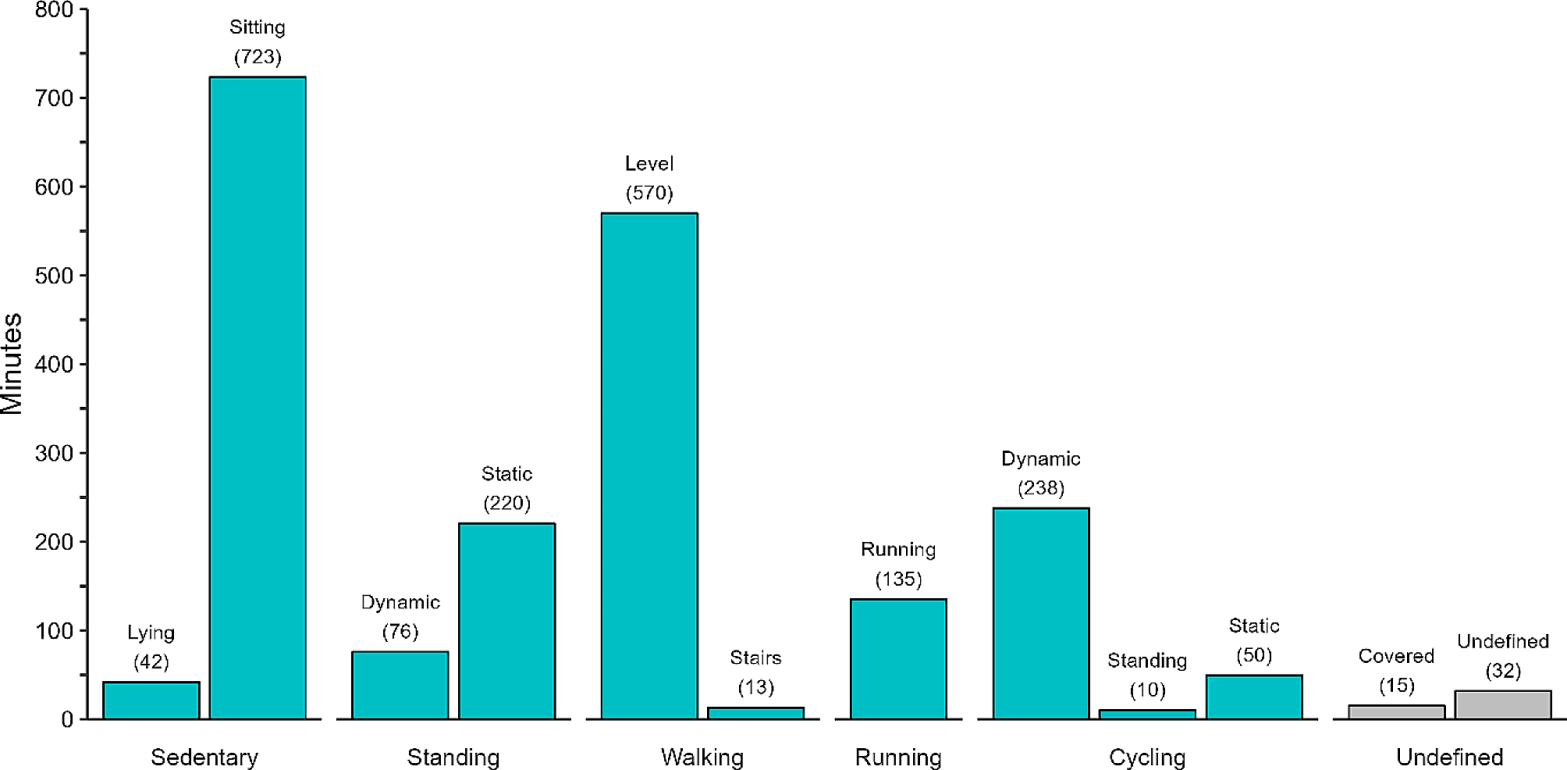
Time distribution of the labelled video data for each primary activity class during free-living condition. Values in parenthesis represent minutes

Labelled activity reference data for a total of 63.6 h were obtained during both conditions. A total time of 34.6 h of video recordings were labelled with the respective activity class for the free-living condition. An additional 32 min of video data (1.5% of the total time) could not be labelled with any activity class (i.e. undefined) and 15 min could not be annotated due to the camera being covered (i.e. due to extra clothing). In both cases, the data were excluded from further analysis. The distribution of each activity class for the free-living condition is shown in Fig. 3. Cohen’s Kappa for the interrater agreement between the two independent raters was 0.95.

Cohen’s Kappa values for the SENS classification and the corresponding reference for the laboratory and free-living condition were 0.97 and 0.86, respectively. Cohen’s Kappa values for the ActiPASS classification were 0.88 and 0.92. The performance metrics for both classification algorithms and the individual activity types are shown in Table 2. In the laboratory condition, mean balanced accuracy ranged from 0.96 to 0.99 for SENS and from 0.82 to 0.99 for ActiPASS. In free-living conditions, mean balanced accuracy for the SENS classification ranged from 0.81 (cycling) to 0.99 (running), while mean balanced accuracy for ActiPASS ranged from 0.92 (walking) to 0.99 (sedentary).

**Table 2:** Classification performance for SENS and ActiPASS classifications during laboratory and free-living conditions

Condition	Algorithm	Activity	Precision	Sensitivity	Specificity	Balanced Accuracy
Laboratory	SENS	Sedentary	0.98 [0.97, 0.99]	1.00 [1.00, 1.00]	0.99 [0.99, 0.99]	0.99 [0.99, 0.99]
		Standing	0.99 [0.99, 1.00]	1.00 [1.00, 1.00]	0.99 [0.99, 1.00]	0.99 [0.99, 1.00]
		Walking	0.93 [0.91, 0.95]	0.97 [0.96, 0.99]	0.98 [0.98, 0.99]	0.98 [0.97, 0.98]
		Running	1.00 [1.00, 1.00]	0.99 [0.99, 0.99]	1.00 [1.00, 1.00]	0.99 [0.99, 0.99]
		Cycling	1.00 [1.00, 1.00]	0.93 [0.90, 0.95]	1.00 [1.00, 1.00]	0.96 [0.95, 0.98]
	ActiPASS	Sedentary	0.99 [0.98, 1.00]	1.00 [1.00, 1.00]	0.99 [0.99, 1.00]	0.99 [0.99, 1.00]
		Standing	0.60 [0.58, 0.62]	0.99 [0.98, 1.00]	0.92 [0.91, 0.92]	0.95 [0.95, 0.96]
		Walking	0.93 [0.89, 0.97]	0.65 [0.62, 0.68]	0.98 [0.97, 0.99]	0.82 [0.80, 0.83]
		Running	0.99 [0.98, 1.00]	0.92 [0.89, 0.96]	0.99 [0.99, 1.00]	0.96 [0.94, 0.98]
		Cycling	1.00 [1.00, 1.00]	0.97 [0.92, 1.00]	1.00 [1.00, 1.00]	0.98 [0.96, 1.00]
Free-living	SENS	Sedentary	0.95 [0.92, 0.98]	0.97 [0.95, 0.99]	0.99 [0.98, 0.99]	0.98 [0.97, 0.99]
		Standing	0.84 [0.78, 0.89]	0.80 [0.76, 0.84]	0.97 [0.95, 0.98]	0.88 [0.86, 0.90]
		Walking	0.76 [0.70, 0.82]	0.95 [0.93, 0.97]	0.90 [0.88, 0.93]	0.93 [0.91, 0.94]
		Running	0.95 [0.89, 1.00]	0.98 [0.96, 0.99]	0.99 [0.99, 1.00]	0.99 [0.98, 0.99]
		Cycling	0.98 [0.97, 0.99]	0.61 [0.54, 0.68]	0.99 [0.99, 0.99]	0.81 [0.77, 0.84]
	ActiPASS	Sedentary	0.95 [0.92, 0.98]	0.99 [0.99, 0.99]	0.99 [0.98, 0.99]	0.99 [0.99, 0.99]
		Standing	0.72 [0.68, 0.76]	0.94 [0.92, 0.96]	0.94 [0.93, 0.96]	0.94 [0.93, 0.95]
		Walking	0.96 [0.94, 0.97]	0.85 [0.81, 0.88]	0.99 [0.98, 0.99]	0.92 [0.90, 0.93]
		Running	0.97 [0.93, 0.99]	0.94 [0.89, 0.98]	0.99 [0.99, 0.99]	0.97 [0.95, 0.99]
		Cycling	0.99 [0.99, 0.99]	0.91 [0.88, 0.94]	0.99 [0.99, 0.99]	0.95 [0.94, 0.97]

**Fig. 4:**
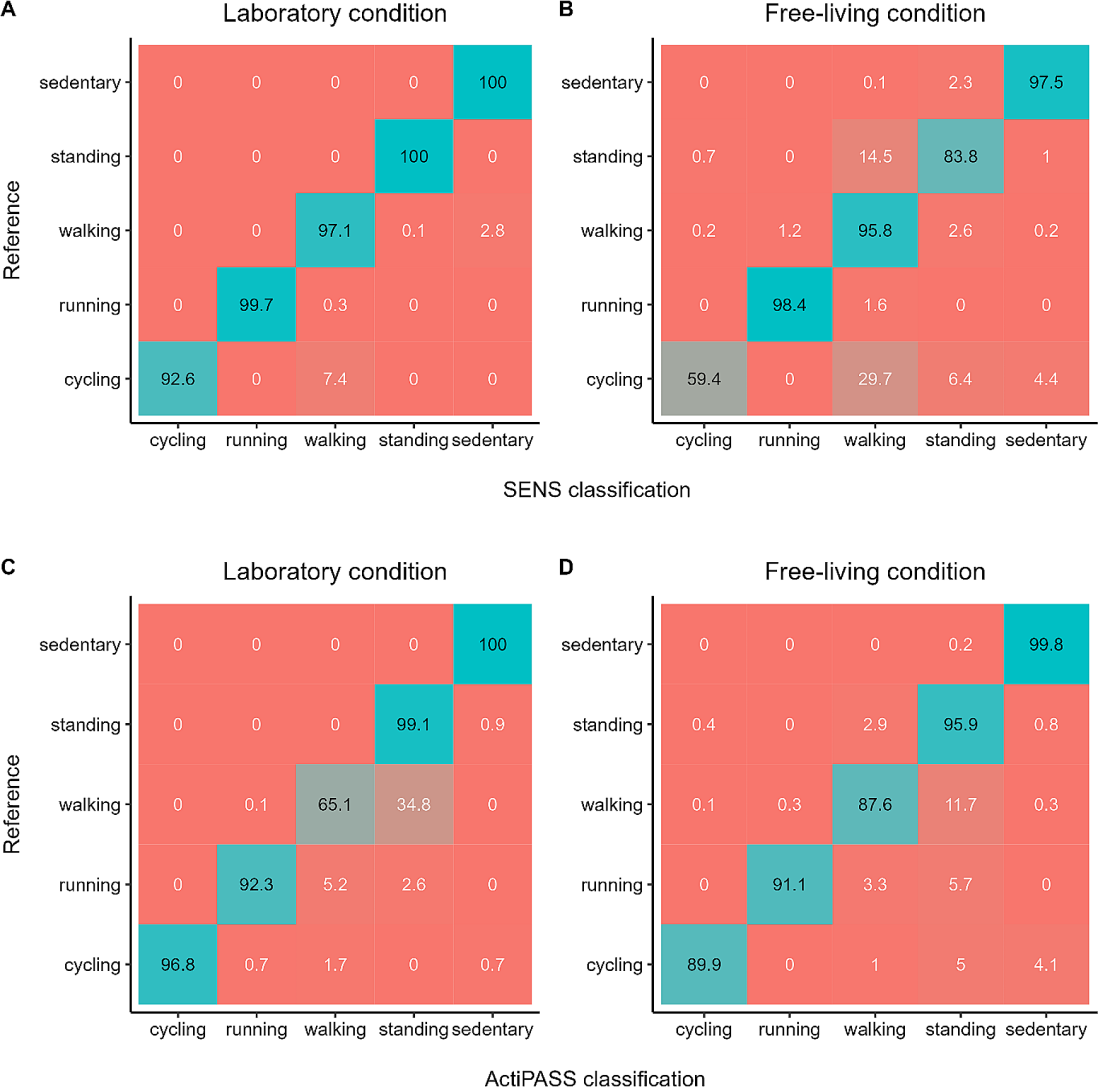
Confusion matrices for SENS and ActiPASS for the laboratory and free-living conditions. **A** and **B** show the SENS motion activity classification during laboratory and free-living conditions, respectively. **C** and **D** show the ActiPASS classification and corresponding reference during laboratory and free-living conditions, respectively. Rows represent the reference activity classes and columns represent the estimated activity classes using the respective classification algorithm. Values represent row percentages

Misclassifications for both classification algorithms and conditions are shown in Fig. 4. The SENS classification algorithm misclassified 7.4% of laboratory cycling and 29.7% of free-living cycling as walking. ActiPASS misclassified 34.8% of laboratory walking as standing. These ActiPASS misclassifications predominantly occurred during slow walking (0.5 m/s), which was misclassified as ‘moving’ (i.e. standing) in 92.9% of all instances of slow walking.

## Discussion

This study aimed to evaluate the performance of two no-code classification methods using the thigh-worn SENS motion accelerometer system during laboratory and free-living conditions. We compared the classification accuracy using the proprietary SENS motion algorithm and classification algorithms implemented in ActiPASS. The classification performance of both methods was highly accurate during laboratory conditions with mean balanced accuracy values ≥ 0.95 for all activity types, except for walking when using ActiPASS. Overall, mean balanced accuracy was lower for all activity types except walking during the free-living condition with values ranging between 0.82 and 0.99 for SENS and 0.92 and 0.99 for ActiPASS. To our knowledge, this is the first study to evaluate the classification accuracy of the SENS motion system (1) during free-living conditions, (2) in a healthy adult population and (3) including cycling as a distinct activity. This study complements ongoing research from the SurPASS project, developing and evaluating a physical behaviour surveillance system based on the SENS motion sensors [13].

Previous studies have investigated the classification accuracy of the SENS motion system in clinical adult populations. Bartholdy et al. [17] reported a 53% mean agreement between SENS and direct observation for classifying sedentary behaviour, standing, walking and miscellaneous activities in adults (*n* = 24) with knee osteoarthritis under laboratory conditions. Pedersen et al. [18] compared the SENS classifications to direct observation in a diverse clinical adult population. For time spent walking and standing, SENS achieved a median agreement (ratio of classified to observed instances) of 88.9% and 67.7%, respectively. However, the exact SENS classification algorithms used for these previous studies may differ from the underlying algorithm within our study due to ongoing development.

The Acti4 algorithm implemented in ActiPASS has been validated to accurately identify sitting, standing, walking, stair walking, running and cycling with specificity ≥ 0.93 and sensitivity ≥ 0.75 for most activity types during semi-standardized and free-living conditions [5, 8, 10]. Notably, the SENS motion accelerometer has a low sampling frequency (12.5 Hz) when compared to other research grade accelerometers that were previously used with the Acti4 algorithm. Therefore, our results highlight a comparable classification performance when using ActiPASS on SENS motion accelerometer data and support the use of ActiPASS across commonly used devices for thigh-worn accelerometry.

Slow walking may be challenging to detect using ActiPASS while SENS accurately classified slow walking as walking. In line with our results, Pedersen et al. [18] reported a high percentage agreement of the SENS system when compared with direct observations for slower walking speed (< 0.67 m/s) in hospitalized patients. Previous studies have reported lower accuracy for the detection of slow walking using other thigh-worn accelerometers [18, 28]. While the precise SENS classification algorithm remains unknow, it seems to consider the acceleration intensity, frequency and symmetry [29]. Given the deterministic design of the Acti4 algorithm used within ActiPASS, it can be inferred that the vertical standard deviation during continuous slow walking did not exceed 0.1 g and was thus classified as ‘moving’ [8]. To our knowledge, no study has yet examined the effect of walking speed on the classification accuracy of ActiPASS or Acti4. The chosen minimum threshold may not be applicable to walking at these low speeds and warrants further investigation.

A recently developed gradient boosting classification algorithm achieved an overall Kappa value of 0.85 using 5-second epochs of thigh acceleration data [9]. Notably, the algorithm achieved a mean sensitivity of 0.90 and mean specificity of 1.00 for free-living cycling. The classification performance during free-living conditions is equally high when compared to ActiPASS classifications. In our study, free-living cycling was not detected as accurately as cycling during laboratory conditions by the SENS algorithm. This may be due to short interruptions of the cyclic movement when the individual stops pedalling (e.g., when doing a turn or cycling downhill). Only 17.1% of the time labelled as static cycling was classified as cycling by the SENS motion classification algorithm. Further post-processing may be necessary to account for these sporadic misclassifications (i.e. by integrating preceding and subsequent activity labels). The ActiPASS classification algorithm performs a mode-based filtering with a windows size of 29 s for cycling and achieved sensitivity values of ≥ 0.93 during outdoor cycling in previous validation studies [5, 8]. In our study, ActiPASS classified free-living cycling with substantially higher sensitivity (mean sensitivity = 0.91) compared to SENS (mean sensitivity = 0.61).

Classification accuracy of both SENS and ActiPASS for overall sedentary behaviour was excellent. It must be noted that the SENS algorithm only classifies overall sedentary behaviour and does not allow more granular analysis of sedentary behaviour types (e.g., differentiating between lying and sitting). Previous research highlights inaccuracies in distinguishing lying and sitting behaviour when using a single thigh-worn accelerometer and machine learning techniques [9, 30]. ActiPASS implements a decision-based algorithm that utilizes the angle of rotation of the thigh during a prolonged lying down period to differentiate sitting from lying [31, 32]. The algorithm was previously validated in a free-living adult working population with a mean sensitivity of 0.94 and specificity 0.95 for lying down. However, these results depend on the posture when lying down. High levels of misclassifications are to be expected in individuals that are less likely to roll over to the side while lying down, for example in populations with low mobility. In our study, only 25% of free-living and 76% of laboratory lying down was correctly classified using ActiPASS (Additional file 2, Figure S1). This may be explained by relatively short time intervals of lying down within our free-living data without much rotation and often supine position. Thus, researchers wanting to differentiate between sitting and lying in positionally constrained individuals or aiming to detect short lying periods may still need to choose a multi-sensor setup to ensure sufficient accuracy [9, 30, 33].

### Strengths, limitations, and considerations

Strengths of the present study comprise the combination of standardised laboratory and unrestricted free-living activities as well as the use of annotated videos as a gold-standard criterion measure for the free-living condition [34]. The range of movement speeds selected for walking, running, and cycling during the laboratory condition integrated slower motions (e.g., 40 rpm while cycling or walking with 0.5 m/s) into the protocol and was thus believed to be more challenging to classify correctly [18]. Our study benefits from the direct comparison of the novel SENS motion activity classification to the established ActiPASS software integrating validated algorithms.

The performance metrics were calculated based on activity groups (i.e. level walking and stair walking were grouped into walking) and did not directly assess the performance for each individual activity class. This approach was chosen to allow a direct comparison of SENS and ActiPASS classification methods. Further, some individual activity labels (e.g., sedentary behaviour with or without movement) could not accurately be distinguished during video annotation. Classification accuracy for individual activity labels (e.g. stair walking) may vary (Additional file 2, Figure S1). The chosen grouping approach affects the performance results in our study. For example, the activity classes ‘sporadic walking’ (SENS) as well as ‘moving’ (ActiPASS) were grouped with ‘standing’ instead of ‘walking’. The choice was made based on the similarity to the video reference label ‘standing dynamic’. According to their definitions, these activity classes describe a participant standing upright with minor movements occurring without purposefully walking [15, 29]. It must be noted that the research field currently lacks a consensus taxonomy for activity types, especially with regards to standing, stepping (or moving) and walking. This currently prohibits a detailed and meaningful analysis of these three individual behaviour types.

The exclusion of any other miscellaneous activity types (1.5% of the total free-living time in our study) for the analysis has an impact on the actual accuracy of both methods during unsupervised activities, as this reduces the number of false positive cases reported in our study. Researchers should be aware, that the accuracy during longer observation periods may be negatively impacted, depending on the prevalence of other miscellaneous activity types outside of the defined activity types.

The study sample was limited to healthy adults aged between 18 and 59 year and thus the results of our study may not reflect the classification performance within other populations such as elderly individuals or clinical populations with impaired movement patterns.

Both SENS and ActiPASS provide additional measures (i.e. step count and activity intensity) as additional outputs. The present study solely focussed on the classification accuracy and no inference to the accuracy of these additional measures can be made.

The specific data processing steps and activity classification algorithm integrated into the SENS motion system currently remain unknown. This presents an important limitation within the research context, as it prohibits any discussion and in-depth understanding of the underlying parameters and algorithm design. A non-proprietary, open-source solution would allow researchers to investigate the influence of different parameter settings and potentially enhance the classification accuracy.

### Future investigations

The ability to remotely manage sensors and access data without the need to visit a research site may enable fully remote study designs using the SENS motion accelerometer system. However, the overall feasibility of such remote studies should be the subject of future investigation. Important issues such as wear time compliance, potential adverse events [12] and usability from the participant’s perspective when applying the sensors at home and unsupervised can be investigated. In particular, in our study we observed sensor detachment in hot and humid outdoor conditions in approximately 5% of participants. This may be a potential concern in larger studies and should be investigated further. The accurate detection of non-wear times is an important processing step in any unsupervised multiday study. ActiPASS allows for manual and automatic non-wear time detection including custom parameter settings as part of its analysis pipeline. To our knowledge, the SENS motion web application currently does not allow for non-wear time detection.

Both classification methods include a predefined range of common activity types that are highly prevalent in the everyday physical behaviour of adults. However, the physical behaviour of free-living adults includes several activity types that are not included in these basic activity types. Future research is needed to better understand the impact of these undefined activity types on classification accuracy and to potentially enable the classification of additional activities (e.g. swimming, climbing, or jumping). In addition, our study protocol ensured accurate placement of the sensor. Variations in placement on the thigh may affect classification accuracy and warrant further investigation.

## Conclusions

We found substantial to near perfect classification performance when using the novel SENS motion system and the validated ActiPASS software based on SENS motion accelerometer data in healthy adults. Users of both classification approaches need to be aware that classification accuracy can vary between algorithms, conditions, and activity types. The accuracy of an algorithm that categorises physical behaviour is also influenced by the underlying definition of that behaviour. The SENS algorithm provided highly accurate classification results for sedentary behaviour, walking, and running. The ActiPASS classification was superior to the SENS algorithm for free-living cycling, whereas it was not as accurate for slow walking under laboratory conditions. Researchers using ActiPASS should be cautious when studying walking in populations with lower walking speeds.

Overall, the results of our study support the use of thigh-worn accelerometers and readily available no-code classification approaches to accurately quantify physical activity types and postures of healthy adults in free-living conditions. Future research should incorporate a more heterogeneous sample and a broader range of activities to allow a more generalisable evaluation of the accuracy of both classification approaches. Given previous validation results with other commonly used brands of accelerometers, our findings support the use of ActiPASS as a generic activity classification software that allows data from different data sources to be pooled [5, 35].

In particular, both software classification methods can be used without any programming experience and are therefore considered to be easily accessible. The accuracy of no-code classification methods combined with wireless sensors can help to reduce the burden on researchers using thigh-worn accelerometer methods. These novel approaches may help to further establish the integration of objective measurements of 24-hour physical behaviour in future research studies.

### Electronic supplementary material

Below is the link to the electronic supplementary material.

Supplementary Material 1

Supplementary Material 2

## Data Availability

The anonymised data generated and analysed for this study are available as open data and can be downloaded from the Zenodo repository [10.5281/zenodo.12704411]. The available data comprises raw acceleration data and activity classification data from laboratory as well as free-living conditions as well as the corresponding reference data. Further, the data includes all custom R code necessary to replicate the results, tables, and figures for this study. Video data used for annotating free-living activities are not available due to data privacy regulations.

## Abbreviations

CSV: Comma-Separated Values
SurPASS: Surveillance of Physical Activity, Sedentary Behaviour, and Sleep
rpm: Revolutions per minute

## References

[1] Dunstan DW, Dogra S, Carter SE, Owen N (2021). Sit less and move more for cardiovascular health: emerging insights and opportunities. Nat Rev Cardiol.

[2] Saunders TJ, McIsaac T, Douillette K, Gaulton N, Hunter S, Rhodes RE (2020). Sedentary behaviour and health in adults: an overview of systematic reviews. Appl Physiol Nutr Metab.

[3] Stevens ML, Gupta N, Inan Eroglu E, Crowley PJ, Eroglu B, Bauman A (2020). Thigh-worn accelerometry for measuring movement and posture across the 24-hour cycle: a scoping review and expert statement. BMJ Open Sport Exerc Med.

[4] Stamatakis E, Koster A, Hamer M, Rangul V, Lee I-M, Bauman AE (2020). Emerging collaborative research platforms for the next generation of physical activity, sleep and exercise medicine guidelines: the prospective physical activity, sitting, and sleep consortium (ProPASS). Br J Sports Med.

[5] Crowley P, Skotte J, Stamatakis E, Hamer M, Aadahl M, Stevens ML (2019). Comparison of physical behavior estimates from three different thigh-worn accelerometers brands: a proof-of-concept for the prospective physical activity, sitting, and sleep consortium (ProPASS). Int J Behav Nutr Phys Act.

[6] Troiano RP, McClain JJ, Brychta RJ, Chen KY (2014). Evolution of accelerometer methods for physical activity research. Br J Sports Med.

[7] Åsvold BO, Langhammer A, Rehn TA, Kjelvik G, Grøntvedt TV, Sørgjerd EP (2023). Cohort Profile Update: the HUNT study, Norway. Int J Epidemiol.

[8] Skotte J, Korshøj M, Kristiansen J, Hanisch C, Holtermann A (2014). Detection of physical activity types using triaxial accelerometers. J Phys Act Health.

[9] Bach K, Kongsvold A, Bårdstu H, Bardal EM, Kjærnli HS, Herland S (2022). A machine learning classifier for detection of physical activity types and postures during free-living. J Meas Phys Behav.

[10] Stemland I, Ingebrigtsen J, Christiansen CS, Jensen BR, Hanisch C, Skotte J (2015). Validity of the Acti4 method for detection of physical activity types in free-living settings: comparison with video analysis. Ergonomics.

[11] Narayanan A, Stewart T, Mackay L (2020). A dual-accelerometer system for detecting human movement in a free-living environment. Med Sci Sports Exerc.

[12] Petersen TL, Brønd JC, Benfeldt E, Jepsen R (2022). Integrity and performance of four tape solutions for mounting accelerometry devices: Lolland-Falster Health Study. J Meas Phys Behav.

[13] Crowley P, Ikeda E, Islam SMS, Kildedal R, Schade Jacobsen S, Roslyng Larsen J (2022). The surveillance of physical activity, sedentary behavior, and sleep: protocol for the development and feasibility evaluation of a novel measurement system. JMIR Res Protoc.

[14] Albrecht BM, Flaßkamp FT, Koster A, Eskofier BM, Bammann K (2022). Cross-sectional survey on researchers’ experience in using accelerometers in health-related studies. BMJ Open Sport Exerc Med.

[15] Hettiarachchi P, Johansson P, ActiPASS. Zenodo; 2023 [Accessed 30 Aug 2023]. https://zenodo.org/record/7701098.

[16] Eghøj M, Rossen Møller S, Kildedal R, Rasmussen M, Brandt Petersen M, Gupta N, et al. Fysisk Aktivitet, stillesiddende adfærd og søvn - resultater fra monitorering med accelerometre i danskernes sundhed 2023. Statens Institut for Folkesundhed; 2024.

[17] Bartholdy C, Gudbergsen H, Bliddal H, Kjærgaard M, Lykkegaard KL, Henriksen M (2018). Reliability and construct validity of the SENS motion^®^ activity measurement system as a tool to detect sedentary behaviour in patients with knee osteoarthritis. Arthritis.

[18] Pedersen BS, Kristensen MT, Josefsen CO, Lykkegaard KL, Jønsson LR, Pedersen MM. Validation of two activity monitors in slow and fast walking hospitalized patients. Rehabil Res Pract. 2022;2022:9230081. 10.1155/2022/9230081 PMC9126721 35615755

[19] Milther C, Winther L, Stahlhut M, Curtis DJ, Aadahl M, Kristensen MT, et al. Validation of an accelerometer system for measuring physical activity and sedentary behavior in healthy children and adolescents. Eur J Pediatr. 2023;182:3647–3639. 10.1007/s00431-023-05014-z PMC10460328 37258775

[20] Thomas S, Reading J, Shephard RJ (1992). Revision of the physical activity readiness questionnaire (PAR-Q). Can J Sport Sci.

[21] R Core Team. R: A language and environment for statistical computing. Vienna, Austria: R Foundation for Statistical Computing. 2023. Available from: https://www.r-project.org.

[22] Chang W, Cheng J, Allaire JJ, Sievert C, Schloerke B, Xie Y, et al. Shiny: web application framework for R. 2023. https://CRAN.R-project.org/package=shiny.

[23] Van Hees VT, Fang Z, Langford J, Assah F, Mohammad A, Da Silva ICM (2014). Autocalibration of accelerometer data for free-living physical activity assessment using local gravity and temperature: an evaluation on four continents. J Appl Physiol.

[24] The National Research Centre for the Working Environment (NFA). Acti4. 2024. https://github.com/motus-nfa/Acti4. Accessed 11 Apr 2024.

[25] ELAN, Nijmegen. Max Planck Institute for Psycholinguistics, The Language Archive; 2022. https://archive.mpi.nl/tla/elan.

[26] Cohen J (1960). A coefficient of agreement for nominal scales. Educ Psychol Meas.

[27] Gamer M, Lemon J, Fellows I, Signh P. irr: various coefficients of interrater reliability and agreement. 2019. https://CRAN.R-project.org/package=irr.

[28] Stansfield B, Hajarnis M, Sudarshan R (2015). Characteristics of very slow stepping in healthy adults and validity of the activPAL3^™^ activity monitor in detecting these steps. Med Eng Phys.

[29] SENS motion. Description of activity categories. https://support.sens.dk/hc/en-us/articles/15580259064093-Description-of-activity-categories. Accessed 28 June 2024.

[30] Stewart T, Narayanan A, Hedayatrad L, Neville J, Mackay L, Duncan S (2018). A dual-accelerometer system for classifying physical activity in children and adults. Med Sci Sports Exerc.

[31] Lyden K, John D, Dall P, Granat MH (2016). Differentiating sitting and lying using a thigh-worn accelerometer. Med Sci Sports Exerc.

[32] Hettiarachchi P, Aili K, Holtermann A, Stamatakis E, Svartengren M, Palm P (2021). Validity of a non-proprietary algorithm for identifying lying down using raw data from thigh-worn triaxial accelerometers. Sensors.

[33] O’Brien MW, Daley WS, Schwartz BD, Shivgulam ME, Wu Y, Kimmerly DS (2023). Characterization of detailed sedentary postures using a tri-monitor ActivPAL configuration in free-living conditions. Sensors.

[34] Keadle SK, Lyden KA, Strath SJ, Staudenmayer JW, Freedson PS (2019). A framework to evaluate devices that assess physical behavior. Exerc Sport Sci Rev.

[35] Ahmadi MN, Blodgett JM, Atkin AJ, Chan H-W, Del Pozo Cruz B, Suorsa K (2024). Relationship of device measured physical activity type and posture with cardiometabolic health markers: pooled dose–response associations from the prospective physical activity, sitting and sleep consortium. Diabetologia.

