# Supplementary material for: Thigh-worn accelerometry: a comparative study of two no-code classification methods for identifying physical activity types
Source: Int J Behav Nutr Phys Act. 2024 Jul 17;21:77. doi: 10.1186/s12966-024-01627-1 (PMC11253440; doi:10.1186/s12966-024-01627-1)
Supplement: Supplementary file 1 — Supplementary Material 1 [file 12966_2024_1627_MOESM1_ESM.docx]

**Supplementary Tables S1-S2**

**Table S1:** Description of the individual activity labels and their respective activity group during analysis.

| **Grouped activity label** | **SENS motion  activity label** | **ActiPASS  activity label** | **Video annotation label** |
| --- | --- | --- | --- |
| Sedentary | - Lying or sitting (rest) - Lying or sitting (movement) | - Lying - Sitting | - Lying down - Sitting |
| Standing | - Standing - Sporadic walking | - Standing - Moving | - Standing (static) - Standing (dynamic) |
| Walking | - Walking - Moderate intensity | - Walking - Walking stairs | - Walking - Stair walking |
| Running | - Running or high intensity | - Running | - Running |
| Cycling | - Cycling | - Cycling | - Cycling (static) - Cycling (dynamic) - Cycling (standing) |

**Table S2:** Coding scheme used for video annotation of the free-living video recordings.

| **Activity label** | **Definition** |
| --- | --- |
| Lying down | The person lies down on the stomach (prone), one the back (supine), on the right or left side. Adjusting or changing the body position after lying down is allowed. Movement of arms is allowed. Movement of the feet and legs is allowed if it does not lead to a change in the posture. |
| Sitting | The person’s buttocks are in contact with the seat of a chair, bed, floor etc. Sitting can include movement of the upper body and legs. Adjustment of sitting position is allowed. |
| Standing  (static) | Upright position. The feet are supporting the person’s body weight without feet movement. Static standing can be inferred if the position of the feet is equal before and after movement of the upper body where the view of the feet disappears on video. If upper body and surroundings indicate no feet movement, standing can be inferred although the feet are not visible. |
| Standing (dynamic/shuffling) | Stepping in place by non-cyclical and/or non-directional leg movements. This includes turning on the spot with leg/feet movement not as part of walking. When not able to see the feet on video (e.g. while standing next to a table/desk), if movement of the upper body and surroundings indicate non-directional feet movement, shuffling can be inferred. |
| Walking | Directional locomotion with at least one stride (i.e., heel-off to heel-strike) and moving of the centre of gravity. Walking can occur in all directions including backwards. |
| Stair walking | Overcoming stairs with at least two steps by bipedal walking. Stair walking starts with the heel-strike on the first step of the stairs. Stair walking ends with the first heel-strike on a flat floor/surface. Stair walking includes short periods of 1-3 seconds between two larger sets of stairs. |
| Running | Directional locomotion with at least two concurrent steps where both feet leave the ground simultaneously. Running can be inferred when trunk moves forward in a constant upward-downward motion with at least two steps. Running along a curved line is allowed. Running backwards is allowed. |
| Cycling  (static) | The person is sitting with the buttocks placed on the seat without any pedalling motion. The bike and person are moving relative to the surrounding. Static cycling ends when the bike is still and the first foot is on contact with the ground or when the person starts pedalling. |
| Cycling  (dynamic) | The person is pedalling while the buttocks are placed on the seat. Dynamic cycling starts with the first pedalling motion. Dynamic cycling ends either (1) when the bike is still and the first foot is on contact with the ground, (2) when the person stops pedalling but continues to move with the bike or (3) when the person stands up while cycling. |
| Cycling  (standing) | The person is standing with both feet on the pedals. Standing cycling starts when the buttocks leave the seat and ends when the buttocks are in contact with the seat. |
| Undefined | The person's activity cannot be classified into any of the other categories defined. Mounting the bike (on and off) is classified as "undefined" as well as squatting on the ground, jumping on two legs or a single leg. |

*The scheme is a modified version used in a previous study by Bach et al. [9].*
