# Supplementary material for: Thigh-worn accelerometry: a comparative study of two no-code classification methods for identifying physical activity types
Source: Int J Behav Nutr Phys Act. 2024 Jul 17;21:77. doi: 10.1186/s12966-024-01627-1 (PMC11253440; doi:10.1186/s12966-024-01627-1)

**Supplementary Figure S1**

**Figure S1:** Confusion matrices for the individual activity labels for SENS (A and B) and ActiPASS classifications (C and D) during laboratory and free-living conditions, respectively.


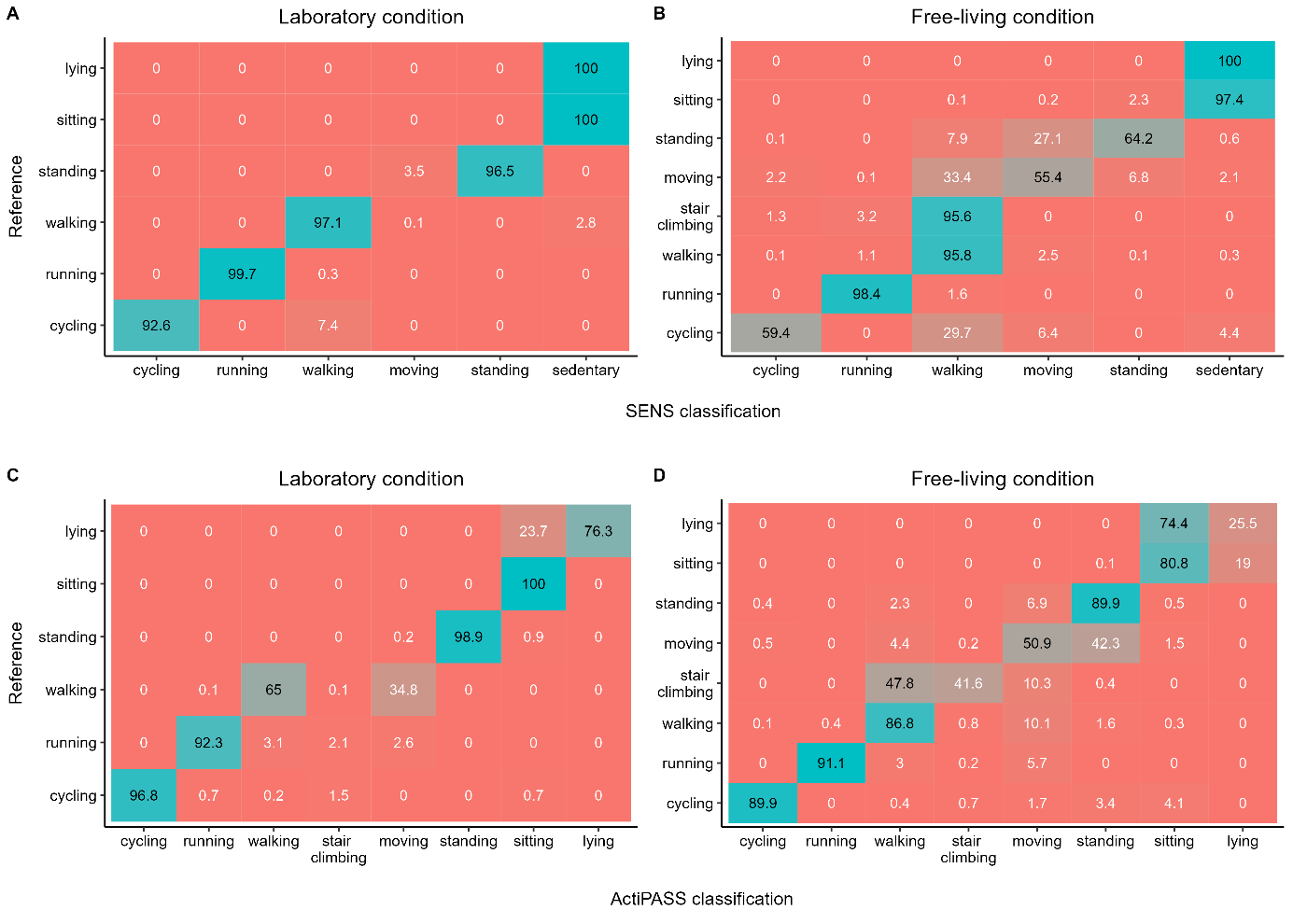

Supplement: Supplementary file 2 — Supplementary Material 2 [file 12966_2024_1627_MOESM2_ESM.docx]
